# Exercise: a “new drug” for elderly patients with chronic heart failure

**DOI:** 10.18632/aging.100901

**Published:** 2016-03-04

**Authors:** Roberto Antonicelli, Liana Spazzafumo, Simonetta Scalvini, Fabiola Olivieri, Maria Vittoria Matassini, Gianfranco Parati, Donatella Del Sindaco, Raffaella Gallo, Fabrizia Lattanzio

**Affiliations:** ^1^ Department of Cardiology, INRCA-IRCCS National Institute, Ancona, Italy; ^2^ Biostatistics Centre, INRCA-IRCCS National Institute, Ancona, Italy; ^3^ Department of Cardiology, Maugeri Foundation IRCCS, Lumezzane (BS), Italy; ^4^ Department of Clinical and Molecular Sciences, DISCLIMO, Università Politecnica delle Marche, Ancona, Italy; ^5^ Center of Clinical Pathology and Innovative Therapy, INRCA-IRCCS National Institute, Ancona, Italy; ^6^ Clinic of Cardiology, Università Politecnica delle Marche, Ancona, Italy; ^7^ Department of Cardiovascular, Neural and Metabolic Sciences, S. Luca Hospital, Italian Auxology Institute, Milano, Italy; ^8^ Department of Health Sciences, University of Milano-Bicocca, Milan, Italy; ^9^ Department of Cardiology, INRCA-IRCCS National Institute, Roma, Italy; ^10^ Department of Cardiology, INRCA-IRCCS National Institute, Cosenza, Italy; ^11^ Scientific Direction, INRCA-IRCCS National Institute, Ancona, Italy

**Keywords:** physical exercise, chronic heart failure, quality of life, elderly CHF patients, 6MWT, QoL

## Abstract

Patients with chronic heart failure (CHF) experience progressive deterioration of functional capacity and quality of life (QoL). This prospective, randomized, controlled trial assesses the effect of exercise training (ET) protocol on functional capacity, rehospitalization, and QoL in CHF patients older than 70 years compared with a control group. A total of 343 elderly patients with stable CHF (age, 76.90±5.67, men, 195, 56.9%) were randomized to ET (TCG, n=170) or usual care (UCG, n=173). The ET protocol involved supervised training sessions for 3 months in the hospital followed by home-telemonitored sessions for 3 months. Assessments, performed at baseline and at 3 and 6 months, included: ECG, resting echocardiography, NT-proBNP, 6-minute walk test (6MWT), Minnesota Living with Heart Failure Questionnaire, and comprehensive geriatric assessment with the InterRAI-HC instrument. As compared to UCG, ET patients at 6 months showed: i) significantly increased 6MWT distance (450±83 vs. 290±97 m, p<0.001); ii) increased ADL scores (5.00±2.49 vs. 6.94±5.66, p=0.037); iii) 40% reduced risk of rehospitalisation (hazard ratio=0.558, 95%CI, 0.326-0.954, p=0.033); and iv) significantly improved perceived QoL (28.6±12.3 vs. 44.5±12.3, p<0.001). In hospital and home-based telemonitored exercise confer significant benefits on the oldest CHF patients, improving functional capacity and subjective QoL and reducing risk of rehospitalisation.

## INTRODUCTION

Congestive heart failure (CHF) is a disabling syndrome affecting 1–2% of the adult western population. Its prevalence increases with age, rising to ≥ 10% among individuals aged ≥ 70 years^1^ and to 50% among those aged ≥ 85 years. The proportion of elderly patients with CHF is set to rise as a consequence of three main factors: extended average life expectancy, enhanced response rates to treatments, and longer survival after acute cardiovascular events [2-4].

One of the main consequences of CHF is progressively impaired functional capacity, which in the elderly is exacerbated by a significant amount of comorbidities. Even though emerging evidence shows that exercise in adult CHF patients is associated with improvements in symptoms, exercise tolerance, and quality of life (QoL), which ameliorate clinical outcomes [5], the approach is still far from widespread. However, the data reported by HF-ACTION (Heart Failure: A Controlled Trial Investigating Outcomes of Exercise Training), describes non-significant reductions in the primary endpoint (all-cause mortality or hospitalization) and in key secondary clinical endpoints [6].

A number of clinical trials have confirmed that evidence-based CHF treatments can improve the prognosis of these patients, however very few studies have investigated patients aged > 70 years; in general, HF patients with common comorbidities such as renal failure, liver disease and cognitive impairment were excluded due to their high morbidity and mortality, high rate of hospitalization, and poor QoL [4]. Similarly, few randomized clinical trials, including a small number of patients assessed the safety and effectiveness of cardiac rehabilitation in CHF patients aged more than 75 years [7-10]. One of the earliest trials involving patients older than 75 years, showed an improvement in exercise tolerance and QoL [11].

A recent meta-analysis of seven prospective randomized controlled trials confirmed that exercise training (ET) does not increase mortality/rehospitalisation rates and improves 6 minute-walk test (6MWT) distance in elderly (> 60 years old, range 70-81 years) CHF patients with reduced systolic function and New York Heart Association (NYHA) functional class II or III. The authors conclude that assessment of ET efficacy in old CHF patients requires further investigation using large, rationally designed controlled clinical trials [12].

Recent studies have demonstrated the value of remote telemonitoring in the management of CHF patients [13-18]. However, the effectiveness of telemonitored home-based cardiac rehabilitation exercise in CHF patients aged more than 75 years is still unclear. Interesting results are expected from an ongoing prospective randomized multicentre study of a novel telemedicine approach [19].

This prospective, controlled trial involved stable CHF patients aged more than 70 years randomized to receive a exercise training program in the hospital for 3 months followed by telemonitored home program for the 3 further months, or usual care.

The primary outcome was the effectiveness of CR program in improving functional performance as assessed by the 6MWT; the secondary endpoints were the effectiveness of CR in preventing hospitalization and in improving perceived QoL and multidimensional geriatric assessment.

## RESULTS

**Figure 1:**
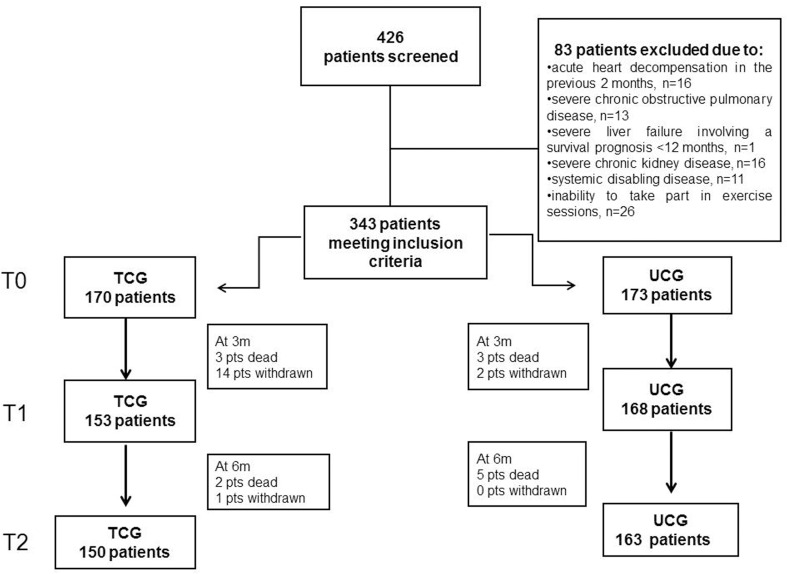
The flow of the 426 consecutive CHF patients enrolled for the study. TCG=training care group; UCG=usual care group.

The flow of the enrolled patients is showed in Figure 1. The 343 patients were randomized to the ET (n=170) and the UC (173) groups. At 3 months (T1), 16 patients dropped out and at 6 months (T2) one more.

13 patients (3.8%) died during the study.

The baseline characteristics of the study population are reported in Table 1.

**Table 1:** Baseline demographic and clinical characteristics of the groups studied

	TCG (n=170)	UCG (n=173)	Total (n=343)	P value
**Age** years, mean±SD	76.21±5.21	77.60±6.02	76.90±5.67	0.145
**Males,** n (%)	103 (60.6%)	92 (53.2%)	195 (56.9%)	0.166
**BMI,** kg/m^2^	26.6±4.4	25.1±5.5	26.8±4.6	0.031
**Systolic arterial pressure,** mmHg	127.1±15.6	124.9±16.2	126.0±16.0	0.423
**Diastolic arterial pressure,** mmHg	71.2±9.4	73.6±11.2	72.3±10.3	0.168
**6MWT**, metres	299±120	270±120	285±121	0.153
**LVEF,** %	47.9±13.3	49.0±13.4	48.4±13.4	0.166
**MLHFQ,** score	42.0±14.9	46.8±16.8	44.3±16.0	0.074
**NT-proBNP,** pg/ml	1236 (2038)^§^	618 (520)^§^	806 (820)^§^	0.110*
**Informal support services**	170 (100%)	156 (90%)	326 (95%)	<0.001

* Variable was log transformed,

§ median (interval).

BMI: body mass index, calculated as weight in kilograms divided by the square of the height in metres (kg/m^2^); LVEF: Left ventricular ejection fraction; 6MWT: Six Minute Walk Test; MLHFQ: Minnesota Living with Heart Failure Questionnaire; NT-proBNP: N-terminal portion of brain-type natriuretic peptide. TCG=training care group; UCG=usual care group. Informal support services: child or child-in-law, other relative, spouse or partner, and friend/neighbour. Continuous data are expressed as mean ± SD and categorical data as number and percentage.

Men accounted for 56.9% of participants; their mean age was 76.90±5.67 years (43% > 75 years).

As regards the cause of CHF, 49% was due to ischemia, 36% to hypertension, and 15% to valve defects. All patients showed significant physical impairment as assessed by the 6MWT (mean 285±121 m at T0) and had mild to moderate left ventricular dysfunction as measured by a mean left ventricular EF (LVEF) of 48.4% (37% with LVEF < 40%).

There were no differences between the two groups at baseline. The mortality rate during the study was 3.8% (n=13).

### Primary endpoint

Functional capacity as measured by 6MWT distance increased significantly in ET at both T1 and T2 (p < 0.001) (Table 2). Moreover in the ET group the walked distance compared with baseline was significantly greater at both time points (p=0.003).

**Table 2:** 6MWT distance in TCG and UCG patients

	TCG	UCG	P value*
**6MWT (metres)**			
**T0**	299±120	270±120	<0.001
**T1**	380.7±120.3	300.6±125.7	
**T2**	394.1±123.6	301.2±125.8	

* ANOVA for repeated measures. T0: baseline; T1: 3-month follow-up; T2: 6-month follow-up; 6MWT: Six Minute Walk Test; MLHFQ: Minnesota Living with Heart Failure questionnaire; NT-proBNP: N-terminal portion of brain-type natriuretic peptide. TCG=training care group; UCG=usual care group. Continuous data are expressed as mean ± SD and categorical data as number and percentage.

### Secondary endpoints

Over the 6 months of the study, 85 patients (13.1%) required an hospitalisation (25 ET and 60 UC). The effect of ET on the risk of rehospitalisation was significant (p < 0.001). The HR, 95% CI, and p values for rehospita-lisation for the two groups are reported in Table 3a.

**Table 3A:** Effect of exercise on hospitalization (Cox model)

	TCG (n=150)	UCG (n=163)	P	HR (95%CI)
**All-causes** **hospitalizations**	25 (15.2%)	60 (36.8%)	<0.001	2.91 (1.70-4.97)

TCG=training care group; UCG=usual care group.

After adjustment for the covariates, ET was found to reduce the risk of all-cause hospitalizations by 44.2% (B coefficient = 0.558, 95%CI, 0.326-0.954, p=0.033) (Table 3b, Figure 2a). Classification tree analysis using the variables of the Cox model, i.e. age, gender, BMI, follow-up 6MWT distance, ADL and IADL scores, and QoL score as dichotomized variables was performed to identify the factors affecting hospitalization (Figure 2b).

**Table 3B:** Effect of exercise on hospitalization, adjusted for clinical covariates

	B	HR	95%CI	P value
**UCG *vs***. **TCG**	0.583	**1.792**	**1.048-3.065**	**0.033**
**Age**	−0.017	0.983	0.932-1.036	0.524
**Gender**	0.129	1.138	0.652-1.985	0.650
**ADL**	−0.003	0.997	0.934-1.063	0.917
**IADL**	0.023	1.023	0.965-1.084	0.446
**BMI**	−0.061	**0.941**	**0.888-0.996**	**0.037**
**6MWT**	−0.002	**0.998**	**0.996-0.999**	**0.044**
**MLHFQ**	0.004	1.004	0.989-1.018	0.630

B= beta coefficient. BMI=body mass index; ADL=activities of daily living. IADL=Instrumental activities of daily living. 6MWT=6 Minute Walk Test; MLHFQ=Minnesota Living with the Heart Failure Questionnaire. HR=hazard ratio. TCG=training care group; UCG=usual care group.

**Figure 2a:**
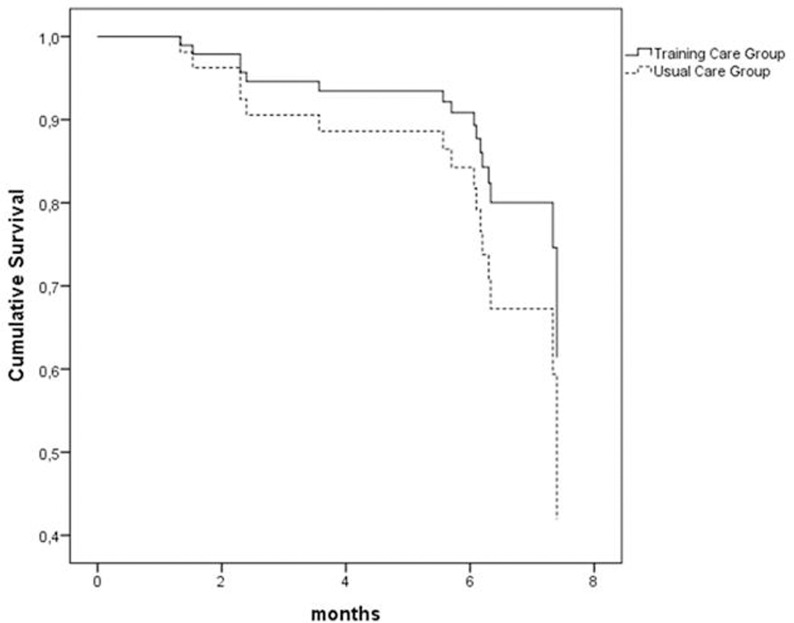
Effect of exercise training and usual care on rehospitalization Cox model adjusted for age, gender, BMI, 6MWT distance, ADL, IADL, and QoL score. TCG=training care group; UCG=usual care group.

**Figure 2b:**
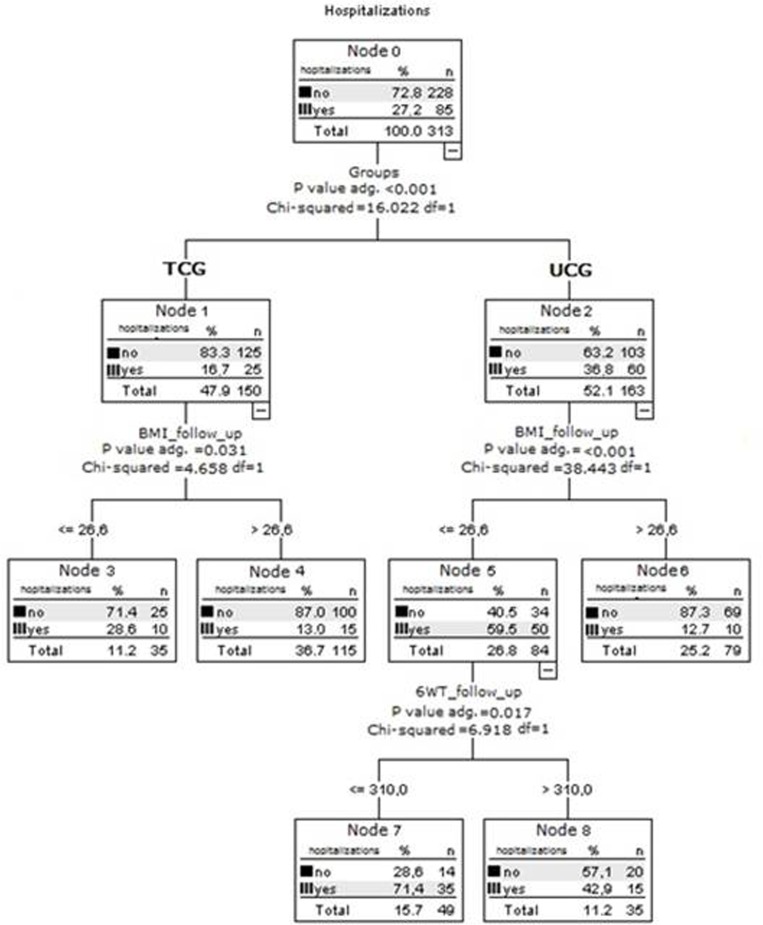
Effect of usual care and exercise training on rehospitalization Decision-tree analysis developed by CHAID (Chi-squared Automatic Interaction Detector) to assess the risk of rehospitalization including dichotomized variables such as age, gender, BMI, 6MWT distance, ADL, IADL and QoL score as predictors. TCG=training care group; UCG=usual care group. P value adj. = p-value of the chi-square test, adjusted by Bonferroni's correction. DF = degree of freedom.

BMI < 26.67 involved an increased risk of rehospitalisation in all CHF patients (Figure 2b).

The InterRAI-HC data are reported in Supplementary Table 1 and show that ADL improved significantly more (p=0.037) in ET than UC participants.

QoL improved significantly in ET compared with UC subjects (p<0.001) (Table 4). Analysis of the respective mean scores showed a comparable perceived QoL at T1 (p=0.987) in both groups, whereas the difference compared with the respective baseline score was significant for both groups. The difference between the groups at T2 (ET, 28.6±12.3; UC, 44.5±12.3) was significant (p<0.001).

**Table 4:** Change in secondary endpoints (MLHFQ and NT-proBNP) at 3 months and 6 months in the two groups

	TCG	UCG	P value*
**MLHFQ**			
**T0**	42.0±14.9	46.8±16.8	**<0.001**
**T1**	29.9±9.8	34.7±9.3	
**T2**	28.6±12.3	44.5±12.3	
**NT-proBNP- pg/ml**			
**T0**	1236 (2038)^§^	618 (520)^§^	**<0.001**
**T1**	350 (137)	290 (241)	
**T2**	440 (208)	2143 (1638)	

* ANOVA for repeated measures. T0: baseline; T1: 3-month follow-up; T2: 6-month follow-up; MLHFQ: Minnesota Living with Heart Failure Questionnaire; NT-proBNP: N-terminal portion of brain-type natriuretic peptide. TCG=training care group; UCG=usual care group.

NT-proBNP was significantly lower (p<0.001) in ET than in UC patients at both time points. The reduction was stronger at T1; however, even though values then rose at T2 in both groups, they remained lower in ET subjects (Table 4).

### Adherence to and safety of exercise training

Overall, 69% of patients attended at least 70% of hospital rehabilitation sessions; the remaining 31% attended at least 50% of sessions.

As regards telemonitored home ET, 77% of patientsperformed at least 70% of sessions; the remaining 23% performed at least 50% of sessions.

ET was safe and well tolerated, and sustained arrhythmia, pre-lipothymia and syncope, angina, traumatic events, or falls never occurred during sessions.

## DISCUSSION

Whereas the benefits of training have widely been documented in large groups of patients with cardiovascular disease, data on CHF patients, especially on elderly patients, are scarce [20].

Our findings provide fresh evidence for the value of ET in elderly subjects with CHF, by demonstrating that also in this subset of CHF patients the benefits of exercise add to those provided by common medications. In our study, functional capacity improved significantly in ET compared with UC patients, with a significantly greater walked distance at both follow-up assessments in the former group. The 95 m distance increase measured at 6 months is much greater than the previously reported minimal important difference (MID) [21, 22]. The improvement was confirmed by a significant increase in ADL scores. Notably, the ET group experienced a significant reduction in the incidence of all-cause rehospitalisation. The latter finding agrees with the conclusions of recent reviews and meta-analyses that highlighted a significant reduction in the rates of hospitalization of CHF patients performing ET [23]. Finally yet importantly, the improved QoL of our elderly CHF patients is interesting and encouraging, considering that poor physical condition equals social isolation and depression.

An interesting and innovative aspect of the present study is the use of telemonitoring for the management of cardiologic rehabilitation in elderly CHF patients. We confirmed our previous results on the effects of home telemonitoring in elderly patients with CHF on rate of hospitalization, compliance with treatment and quality of life, by comparison with a group receiving usual care [22, 25].

In the present study, the walked distance was significantly greater, the QL was improved and NT-proBNP was reduced compared with baseline after in hospital ET phase. Notably, these benefits were maintained also after the further telemonitored home phase ET. These results highlights how even the rehabilitation program for patients with CHF followed by remote monitoring has led to significant results. This is of clinical relevance, since the clinical benefit of physical exercise in CHF management is related to the duration of ET. In this context, the telemonitoring of ET at home could ensure adherence to the rehabilitation program over a long period. In our study to increase compliance at home, a nurse tutor call the patients during the home ET sessions, similar to the hospital sessions, and the quality of each patient's ET was assessed by remote telemonitoring of one lead ECG before and after each session. Taking into account the high prevalence of CHF in elderly people, ET telemonitoring at home could be an efficacy tool to ensure compliance with rehabilitation treatment for CHF patients, especially for the oldest old ones.

It was recently reported that the use of telemonitoring in the management of heart failure appears to lead to similar health outcomes as face-to-face or telephone delivery of care [26]. For an elderly subject who is not autonomous in moving from home to the clinic hospital, telemedicine represent a promising prerogative for activities that should be daily.

### Conclusion

ET programs associated with drug treatment can attenuate many of the deleterious systemic and tissue-specific effects of CHF, particularly in the elderly. Since CHF is a severe public health problem, particularly in the West, due to progressive ageing of the population, associating physical training and drug treatment can provide important therapeutic effects and thus should be recommended to elderly subjects.

The potential value of “eHealth” applications to support self-management of chronic illness is only beginning to be understood. Conceptually, however, two of the most critical components of self-management, such as self-monitoring and feedback, may both be facilitated by telemonitoring technology. Additionally, eHealth technology offers the potential to provide more ubiquitous and constant support for self-monitoring and feedback than has been previously possible, and therefore represent applications useful into the everyday lives of older adults and elderly patients. One fundamental question, however, is whether older adults, those most likely to have multiple chronic health conditions, such as CHF patients, can be taught to use eHealth applications and, beyond that, whether they will use these devices and applications in ways that improve health. Here we provide some evidence that home telemonitoring could represent a promising tool for the rehabilitation programs of the oldest CHF patients, ensuring that the benefits induced by physical activity remain longer in time.

## METHODS

### Population

From January 2011 to January 2014, 426 consecutive patients with CHF were screened and recruited at 6 Italian Cardiologic Rehabilitation Centres. Inclusion criteria were: in-patients or out-patients aged > 70 years; CHF from any cause with reduced or preserved ejection fraction (EF); NYHA functional class ≥ II; and a Mini Mental State Examination score > 24.

Exclusion criteria were a survival prognosis < 6 months: severe uncontrolled diabetes; acute heart de-compensation in the previous 2 months; myocardial infarction in the previous 2 months; severe chronic obstructive pulmonary disease; severe liver failure with a survival prognosis < 12 months; severe chronic kidney disease with a glomerular filtration rate < 15 mL/min/1.73 m^2^; severe disabling systemic disease, severe cognitive impairment, and inability to perform ET.

The study was approved by the INRCA Institute Ethics Board and complies with the principles stated in the Declaration of Helsinki. All patients gave their written informed consent to participate.

Participants were randomized to a usual care group (UCG) or a training care group (TCG) by a nurse who was not involved in recruitment.

### Clinical assessment

Patients were assessed at baseline (T0) and at 3 (T1) and 6 months (T2) by clinical examination, 12-lead ECG, resting echocardiography, serum levels of N-terminal portion of brain-type natriuretic peptide (NT-proBNP), 6MWT, the multidimensional geriatric assessment InterRAI Home Care Assessment System (InterRai-HC) [27] and the self-administered Minnesota Living with Heart Failure Questionnaire (MLHFQ) [28].

### Assessment tools

The InterRAI-HC instrument was used to collect data on medical, psychosocial, and functional abilities and limitations, including activities of daily living (ADL) and instrumental ADL (IADL). The MLHFQ was used to assess QoL on a scale from 0 (best) to 105 (worst).

### N-terminal portion of brain-type natriuretic peptide

Blood for NT-proBNP was drawn in the morning from an antecubital vein after 30 min of bed rest with the patient in supine position. Serum levels were determined by an ElectroChemiLuminescence ImmunoAssay (ECLIA-Cobas, Roche Diagnostics, Rotkreutz, CH). Il NT-proBNP cut off values were: > 125 pg/ml (< 75 years) and > 450 pg/ml (≥ 75 years).

### Exercise training protocol: Hospital/Educational Phase

The ET program consisted of two successive 3-month phases involving supervised training at the hospital (out-patient service) and telemonitored home exercise, respectively. Before discharge, all patients across all centres were educated about heart failure, including advice on daily weights, daily self-measurement of blood pressure, rate of carrying out blood examinations, dietary restrictions, including sodium and fluid, and signs and symptoms of a heart failure decompensation.

In the first phase, patients attended 3 times weekly, 50-min classes under the supervision of a physiotherapist. Each session consisted of warm-up (10 min), exercise (30 min) and cool-down (10 min); exercise involved riding a cycle ergometer with 5 min warm-up, 20 min of intense exercise at 60 rpm, achieving 60-70% of the maximum predicted heart rate, and 5 min cool-down. ET intensity was based on each participant's functional capacity and was adjusted throughout the study.

### Exercise training protocol: Telemonitored Home-Phase

The rehabilitation program consisted in scheduled telephone call from the nurse-tutor who evaluated the presence of signs and symptoms of decompensation and the need of possible treatment changes, after a consultation with the cardiologists. Before starting the rehabilitation program, the patient recorded a 1-lead ECG signal transmitted to the TMC. One-lead ECG recordings were obtained before and within 5 min of session completion. At the end of the training session, a telemonitoring contact with the nurse-tutor was planned in order to transfer all the 1-lead ECG recordings. Compliance was represented by a heart rate at least 20% higher than that measured before training based on the tele-ECG recorded post-exercise.

### Control group

Patients assigned randomly to UCG were referred to their general practitioner. A structured follow-up with the cardiologist at 12 months in the hospital outpatient department and the appointment with the primary care physician within two weeks from the discharge were planned.

### Statistical analysis

Data were analysed using SPSS software, v. 20.0. A p value < 0.05 was considered significant. Continuous data were expressed as mean ± standard deviation (SD) and categorical data as number and percentage. Variables with asymmetric distribution were summarized as medians and interquartile intervals.

Differences between the groups during follow-up were tested by ANOVA for repeated measures. A Cox proportional hazard model was applied to analyse the multivariate effects of the risk factors, identified by ANOVA, on rehospitalisation. Adjusted hazard ratios (HR) and 95% confidence intervals (CI) were calculated to test the direction and strength of the influence of individual factors on event risk and event-free survival.

CHAID (Chi-squared Automatic Interaction Detector) was used to develop a decision-tree analysis to assess the risk of rehospitalisation using all dichotomized variables as predictors. The CHAID algorithm incorporates a sequential merge and split procedure based on chi-square test statistics. The following stopping rules were applied in growing the tree: minimum terminal parental node size, 50 cases, minimum terminal child node size, 25 cases, and α = 0.05 for splitting nodes and merging categories. The convergence criteria for CHAID analysis were Epsilon=0.0010 and 100 maximum iterations before stopping the process.

The sample size of 343 patients provides a power of 80% to detect an effect size of 0.10 on variation of 6MWT distance between the groups using ANOVA for repeated measures with a 5% significance level. This level also holds for the other aims of this project.

## SUPPLEMENTARY TABLE


