# Supplementary material for: Exercise: a “new drug” for elderly patients with chronic heart failure
Source: Aging (Albany NY). 2016 Mar 4;8(5):860–9. doi: 10.18632/aging.100901 (PMC4931840; doi:10.18632/aging.100901)
Supplement: Supplementary file 1 [file aging-08-0860-s001.pdf]

SUPPLEMENTARY TABLE

Supplementary Table 1

InterRAI_HC	UCG T0 (n=173)	TCG T0 (n=170)	*Total* (N. 343)	p value
**Age** (mean±SD)	77.60±6.02	76.21±5.21	76.90±5.67	0.145
**Gender** :				
Male	92 (53.2%)	103 (60.6%)	195 (56.9%)	0.166
Female	81 (46.8%)	67 (39.4%)	148 (43.1%)	
**Marital Status**				
Married	80 (46.2%)	90 (53.0%)	170 (49.5%)	0.344
Never married	14 (8.1%)	7 (4.1%)	22 (6.3%)	
Divorced	6 (3.5%)	7 (4.1%)	12 (3.6%)	
**Education:**				
Illiterate, n (%)	20 (11.6%)	24 (14.2%)	44 (12.8%)	0.385
Primary school, n (%)	92 (53.2%)	73 (42.6%)	165 (48.1%)	
Secondary school, n (%)	41 (23.7%)	53 (31.4%)	94 (27.4%)	
High school, n (%)	11 (6.4%)	10 (5.9%)	21 (6.1%)	
Illiterate, n (%)	9 (5.2%)	10 (5.9%)	19 (5.5%)	
**Where Lived at Time of Refferal**				-
Private Home/ Apt n (%)	170 (100%)	173 (100%)	343 (100%)	
**Who Lived with :**				
lived alone n (%)	34 (20%)	52 (30%)	86 (25%)	0.756
lived with spouse only n (%)	85 (50%)	52 (30%)	137(40%)	
lived with child n (%)	51 (30%)	69 (40%)	120(35%)	
**Where came from**				
Private Home/ Apt with no home care services n (%)	173 (100%)	170 (100%)	343 (100%)	-

Supplementary Table 1 (continue)

InterRAI_HC	UCG T0 (n=173)	TCG T0 (n=170)	UCG T2 (n=173)	TCG T2 (n=170)	p value
**IADL** (mean±SD)	5.1±3.32	4.98±4.59	5.67±5.32	5.18±4.75	0.408
**ADL** (mean±SD)	5.50±4.10	5.45±4.32	5.00±2.49	6.94±5.66	**0.037**
**Cognitive skills for daily decision-making**					
Independent	104 (60%)	108 (64%)	138 (80%)	170 (100%)	0.064
Minimally Impaired	69 (40%)	62 (36%)	35 (20%)	-	
**Memory recall Ability:**					
Independent	121 (70%)	124 (73%)	104 (60%)	170 (100%)	0.208
Minimally Impaired	52 (30%)	46 (27%)	69 (40%)	-	
**Indicators of Delirium:**					
Independent	138 (80%)	124 (73%)	138 (80%)	170 (100%)	0.422
Minimally Impaired	35 (20%)	46 (27%)	35 (20%)	-	
**Making self understood (Expression):**					
Yes	121 (70%)	139 (82%)	130 (75%)	170 (100%)	0.626
No	52 (30%)	31 (18%)	43 (25%)	-	
**Ability to understand others (comprehension):**					
Yes	138 (80%)	155 (91%)	138 (80%)	170 (100%)	0.350
No	35 (20%)	15 (9%)	35 (20%)	-	
**Hearing:**					
Hears adequately	138 (80%)	139 (82%)	140 (81%)	141 (83%)	0.998
Minimal difficulty	35 (20%)	31 (18%)	33 (19%)	29 (17%)	
**Vision:**					
Adequate	138 (80%)	143 (84%)	151 (87%)	146 (86%)	0.683
Impaired	35 (20%)	27 (16%)	22 (13%)	24 (14%)	

Supplementary Table 1 (continue)

**Mood and Bevihavior Patters**					
Indicators not exhibited in the last 3 days	121 (70%)	112 (66%)	104 (60%)	128 (75%)	0.634
Exhibited 1-2 of last 3 days	52 (30%)	58 (34%)	69 (40%)	43 (25%)	
**Bladder Continence**:					
Continent	152 (88%)	155 (90%)	152 (88%)	170 (100%)	0.909
Incontinent	22 (12%)	15 (10%)	17 (12%)	-	
**Bowel Continence**:					
Continent	173 (100%)	170 (100%)	173 (100%)	170 (100%)	-
**Falls**:					
no	173 (100%)	161 (95%)	156 (90%)	170 (100%)	-
yes	-	9 (5%)	17 (10%)	-	
**Intensity of Pain:**					
no	52 (30%)	77 (45%)	128 (74%)	170 (100%)	<0.001
daily: less than 4 hours	104 (60%)	77 (45%)	45 (26%)	-	
daily: at least every 2-4 hours	17 (10%)	15 (10%)	-	-	
**Medications adequately control pain**:					
yes	173 (100%)	155 (91%)	173 (100%)	170 (100%)	0.348
no	-	15 (9%)	-	-	
**Nutritionand Oral Status**					
Adequate	156 (90%)	162 (95%)	147 (85%)	170 (100%)	0.426
Not Adequate	17 (10%)	9 (5%)	26 (15%)	-	
**Skin Condition**					
No Skin problems	138 (80%)	132 (78%)	121 (70%)	149 (88%)	0.530
Skin problems	35 (20%)	38 (22%)	52 (30%)	21 (12%)	
**Compliance/ Adherence with medications**					
always compliant	173 (100%)	170 (100%)	173 (100%)	170 (100%)	-
